# Inter and Intra Rater Reliability of the 10 Meter Walk Test in the Community Dweller Adults with Spastic Cerebral Palsy

**Published:** 2017

**Authors:** Fariba BAHRAMI, Shohreh NOORIZADEH DEHKORDI, Mehdi DADGOO

**Affiliations:** 1 Department of Physiotherapy, Iranian center of excellence in Physiotherapy, School of Rehabilitation Sciences, Iran University of Medical Sciences, Tehran, Iran

**Keywords:** Reliability, 10 meter walk test, Walking speed, Cerebral palsy

## Abstract

**Objective:**

We aimed to investigation the intra-rater and inter-raters reliability of the 10 meter walk test (10 MWT) in adults with spastic cerebral palsy (CP).

**Materials & Methods:**

Thirty ambulatory adults with spastic CP in the summer of 2014 participated (19 men, 11 women; mean age 28 ± 7 yr, range 18- 46 yr). Individuals were non-randomly selected by convenient sampling from the Ra’ad Rehabilitation Goodwill Complex in Tehran, Iran. They had GMFCS levels below IV (I, II, and III). Retest interval for inter-raters study lasted a week. During the tests, participants walked with their maximum speed. Intraclass correlation coefficients (ICC) estimated reliability.

**Results:**

The 10 MWT ICC for intra-rater was 0.98 (95% confidence interval (CI) 0.96-0.99) for participants, and >0.89 in GMFCS subgroups (95% confidence interval (CI) lower bound>0.67). The 10 MWT inter-raters’ ICC was 0.998 (95% confidence interval (CI) 0/996-0/999), and >0.993 in GMFCS subgroups (95% confidence interval (CI) lower bound>0.977). Standard error of the measurement (SEM) values for both studies was small (0.02< SEM< 0.07).

**Conclusion:**

Excellent intra-rater and inter-raters reliability of the 10 MWT in adults with CP, especially in the moderate motor impairments (GMFCS level III), indicates that this tool can be used in clinics to assess the results of interventions.

## Introduction

One of the factors that restrict social participation of adults with cerebral palsy (CP) is the gait disorder (1). Gait disorders in patients with CP are due to several factors including spasticity (2), reduced muscle strength (3), decreased range of motion (4) and decrease in walking speed and endurance (5). 

One of the key indicators to measure the performance in neurological diseases is the walking speed (6). Social participation, community ambulation and falling risk can be estimated by measuring the gait speed (7, 8). Among various tools used to measure the gait speed clinically is the 10 Meter Walk Test (10 MWT). This test has high validity in the neurological diseases (9). It can be completely safe and easily used with minimal facilities and budget (9). 

The 10 MWT can be done in two different ways, one with comfortable gait speed and the other with fast walking speed. Both have good to excellent reliabilities in adults (coefficient ≥ 0.903) (10) and in children with neurological diseases (for comfortable gait speed, ICC has ranges between 0.91 to 0.97, and for fast gait speed it falls in the range of 0.70 to 0.87) (11). Average speed of the 20 to 40 years healthy adults ranges from 2.462 to 2.533 meters per second in men and from 2.123 to 2.467 meters per second in women (10). 

Many studies reported excellent intra and inter-rater reliability of the 10 MWT in the neurological diseases such as Parkinsonism, children with neuromuscular disease, spinal cord injuries, stroke and traumatic brain injury (ICC ≥ 0.87) (12-18). However, there are a few methodological studies on the reliability of this test in patients with CP. Test-retest reliability of the 10 Meter Fast Walk test (10 MFWT) was evaluated in the ambulatory CP children, with subgroup analyses in Gross Motor Function Classification System (GMFCS) levels of I, II, and III. The ICC of this test was 0.81 among participants, and over 0.59 in GMFCS subgroups. Accordingly, the fast 10 MWT showed inadequate test– retest reliability (19). 

The aim of this study was to examine the reliability of the intra-rater and inter-raters of 10 MFWT in adult CP and in GMFCS subgroups (levels I, II , and III). 

## Material & Methods

In this cross-sectional study, 30 adults with spastic CP (19 men and 11 women; mean age 27 yr and 9 months, SD 7 yr) in the summer of 2014 were recruited. Individuals were non-randomly selected by convenient sampling from the Ra’ad Rehabilitation Goodwill Complex in Tehran, Iran. After obtaining permission from the Research Ethics Committee with license no. 94/105/58, informed consent was obtained from participants. Demographic data and personal information were collected from the patients’ records. 

**Participants**

Individuals were included provided they had a diagnosis of spastic CP confirmed by a neurologist with CT scan. They were over 18 yr, and had ability to follow verbal commands and to walk with or without assistive devices (walkers or various types of canes). Our participants had GMFCS levels below IV (mild to moderate motor impairments). According to the GMFCS, people at levels I and II are called as mild motor impairment. Individuals at level III, are recognized as moderate motor impairment. Additionally, severe motor impairment are located in levels IV and V (20). People who were wheelchair-bounded and staying at the sanitarium, or they had lower limbs orthopedic surgery or the botulinum toxin injection in the last six months prior to their participation, or had heart disease or uncontrolled seizures were excluded. Besides, if a volunteer had any kinds of physical fitness, sports, or physical therapy at one-week interval between test and retest he/she would be excluded.

**Procedures**

For intra–rater reliability one physiotherapist and for inter–rater reliability two physiotherapists with 12 yr experience of working with CP were recruited. At first, participants were familiarized with the procedure of fast 10 MWT. Measurements were performed between 9 to 11 am. Individuals, who used the assistive device for walking, were asked to utilize the same device during the 10 MWT. The digital stopwatch with an accuracy of hundredth of a second was used to record the time (5). A mid–stretch of at least 14 m length of a 34 m quiet corridor was set for the walking course. The start and stop lines with two meters plus were demarcated by the colored tape on the floor. The zero and 14-m tracks were marked with green tape, and the 2-m and 12-m tracks with red tape. Two meters plus at the beginning and end of the course was considered to remove acceleration or deceleration (9, 21). 

Before the test, the subjects were warned not to run. Participants were asked to walk with their fastest speed after hearing the Go command and continue down the corridor until told to stop. During the tests, however, the examiner did not encourage the volunteers to increase the speed. Upon passing the leading foot from the first red line, the digital stopwatch was started and once the leading foot crossed the terminal red line, the timer was interrupted (5). By passing the second green line, he/ she stopped after hearing the stop command. The tests were repeated three times in each session. Retest interval lasted a week. For inter–rater reliability test, the examiners were standing in the middle of the 14-m course at opposite side of each other, so that besides viewing the start and end line of the 10-m course, they were unaware of the co-worker’s recorded results. Then, the samples were given two min to rest. The mean of three replications was considered as the time of the test in seconds, and the speed of each individual based on meters per second was achieved. A week later retest was done in the same way by a physiotherapist.

**Statistical Analysis**

The statistical analyses were performed using the SPSS version 16.0 (Chicago, IL, USA). Intra class correlation coefficients (ICC; type 2:1) (23) and associated 95% confidence intervals (CI) estimated the reliability. While ICCs from 0.70 to 0.79 reflect good reliability, an ICC ≥ 0.80 reflects excellent reliability (22). Paired t-tests were used to assess the changes in scores from test to retest and the inter-observer’s scores, and to search for differences within GMFCS levels, I, II and III between the tests. The level of statistical significance was set at P= 0.01. The Bland-Altman method evaluated measurements bias. The mean speed differences of GMFCS subgroups is the estimated bias and the standard deviation (SD) of the speed differences measures the random fluctuations around this mean (22). The standard error of measurement (SEM) indicates that how much of the variation is due to the variation of the mean and how much is related to the nature of the variables (23). SEM and MDC (22) were calculated by these formulas SEM = SD1 × (√1-ICC) (SD1 is the standard deviation of first test scores) MDC95 (95% CI) = 1/96 × √2 × SEM 

## Results

**Participant Characteristics**

The participant’s ages ranged from 20 to 46 yr. Eight volunteers used walkers, four of them used two crutches, one person had one crutch and 17 participants walked without any walking aids. Proportion of the number of men compared with women, in total and in the subgroups (I, II, III) of GMFCS, was higher. Personal information and the number of different types of spastic cerebral palsy including diplegia, hemiplegia, double hemiplegia of total participants and subgroups of GMFCS is given in Table 1. 

**Intra-Rater Reliability**

**Fig 1:**
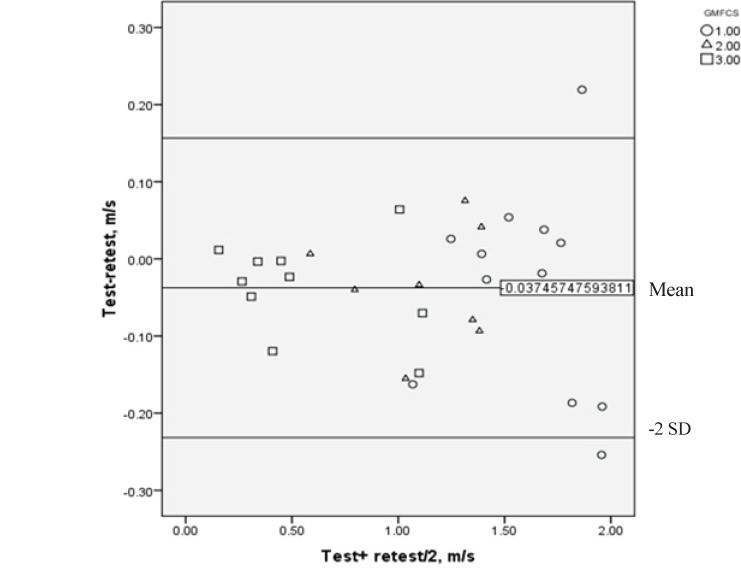
The Bland-Altman plot of distribution of the difference between walking speed measured in two stages of intrarater in subgroups of GMFCS.

Intra-rater changes of the walking speed of the individuals with spastic CP are presented in Table 2. Walking speed changes between the two testing sessions among participants of levels I, II and III of GMFCS was not significant. Intra class correlation coefficients (ICC) of participant’s test-retest walking speeds was 0.98 and ICC of levels I to III of the GMFCS had ranges from 0.89 in level I up to 0.98 in level III, respectively. Table 3 shows ICCs, the lower and upper bounds of the 95% CI, the SEM and the MDC. The Bland–Altman plot (24) (Figure 1) shows distribution of the differences between walking speeds measured in two-stages of test-retest in 30 adults with CP in GMFCS’ subgroups (I, II, III).

**Inter-Raters Reliability**

**Fig 2:**
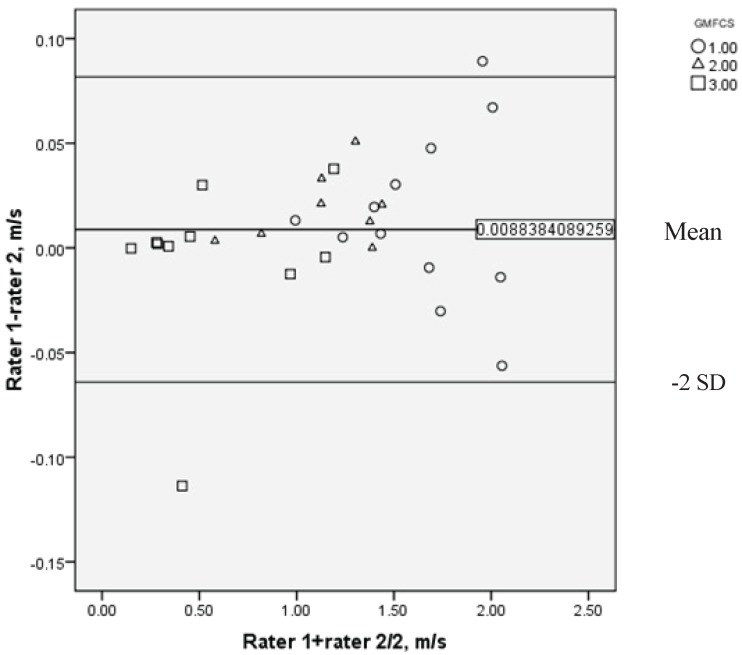
The Bland-Altman plot for distribution of the difference between walking speed measured in two stages of inter-raters in subgroups of GMFCS.

Inter-raters changes of the walking speed of the adults with mild and moderate spastic cerebral palsy are given in Table 4. The walking speeds measured by two therapists did not change significantly. ICC between the raters for all participants was 0.99 with lower and upper bounds of 0.996 to 0.999, respectively. More information of inter observer’s ICC, including GMFCS subgroups data, is provided in Table 5. The Bland–Altman plot (Figure 2) indicates the interrater velocity difference dispersion in 30 adults with CP in GMFCS’ subgroups (I, II, III). The scattering plots in both figures (Figure 1 and 2) were close to the mean line, which is close to zero, and between the ± 2SD lines. There were some outliers. These outliers are included in the calculation, and the reliability statistics are still excellent. These figures also demonstrate that the walking speed in CP patients coincide with the severity of motor disorders, meaning that as the GMFCS level goes up, the walking speed decreases. Irregular dispersions on the positive and negative sides of the mean line in Figure 1 and 2 indicate the absence of training. The plots related to the GMFCS level I in comparison with other levels in this study, indicate more distribution and orientation from the mean line.

**Table 1:** Characteristics of Adults with Spastic Cerebral Palsy

	**All participants**	GMFCS* 1	**GMFCS** * ** 2**	**GMFCS** * ** 3**
**Total (male, female)**	30 (19, 11)	12 (7, 5)	8 (5, 3)	10 (6,4 )
**Mean Age (SD) (yr)**	27.94 (7.05)	25.61 (5.93)	26.53 (3.95)	31.85 (8.91)
**Age range**	20- 46.01	20. 09-39	20-32.09	22.10-46. 01
**Mean height (SD) (m)**	1.62 (1.05)	1.66 (1.15)	1.61 (8.86)	1.58 (9.6)
**Mean Weight (SD) (kg)**	59.86 (11.10)	59.75 (8.32)	59.75 (8.32)	59.4 (1.36)
**Diagnosis**				
**Diplegia**	16	1	6	9
**Hemiplegia**	10	10	0	0
**Double Hemiplegia**	2	0	1	1
**Other**	2	1	1	0

* GMFCS. Gross Motor Function Classification System classified patients with cerebral palsy according to the severity of motor impairment into five categories. Subgroups I and II (mild), III (moderate) and subtypes IV and V (severe)

**Table 2:** Mean (M), Standard Deviation (SD) and Range of Intra- Rater Observer Measurements for 10 MWT (in m/s) in Adults with Mild and Moderate Spastic CP

	**n**	**Mean(SD)** **Test**	**Range test**	**Mean(SD) Retest**	**Range retest**	**Significant**
**All participant**	30	1.15 (0.55)	0.15-2.08	1.11 (0.54)	0 16-1.97	0.04
**GMFCS** * **level I**	12	1.63 (0.28)	1.15-2.08	1.59 (0.30)	0.99-1.97	0.34
**GMFCS** * **level II**	8	1.13 (0.30)	0.58-1.43	1.10 (0.29)	0.59-1.41	0.21
**GMFCS** * **level III**	10	0.58 (0.35)	0.15-1.17	0.54 (0.37)	0.16-1.08	0.11

* GMFCS. Gross Motor Function Classification System

**Table 3:** Intra Class Correlation Coefficient (ICC) and Standard Error of Measurement (SEM) for Intra-Rater Observer 10 MWT in Adults with Spastic CP

	**n**	**ICC**	**95% CI of ICC**	**SEM**	**MDC**
**All participant**	30	0.98	0.96-0.99	0.07	0.18
**GMFCS** * **level I**	12	0.89	0.67-0.96	0.09	0.24
**GMFCS** * **level II**	8	0.97	0.86-0.99	0.05	0.13
**GMFCS** * **level III**	10	0.98	0.93-0.99	0.04	0.11

* GMFCS. Gross Motor Function Classification System

**Table 4:** Mean (M), Standard Deviation (SD) and Range of Inter-Rater Observers Measurements for 10 MWT (in m/s) in Adults with Mild and Moderate Spastic CP

	**n**	**Mean (SD)** **observer 1**	**Range observer 1**	**Mean (SD) observer 2**	**Range observer 2**	**Significant**
**All participant**	30	1.15 (0.57)	0.15-2.04	1.15 (0.56)	0.15-2.08	0.19
**GMFCS** * **level I**	12	1.65 (0.34)	1.0-2.04	1.63 (0.34)	0.99-2.08	0.21
**GMFCS** * **level II**	8	1.15 (0.30)	0.58-1.45	1.13 (0.30)	0.58-1.43	0.03
**GMFCS** * **level III**	10	0.57 (0.38)	0.15-1.21	0.57 (0.37)	0.15-1.17	0.82

GMFCS*: . Gross Motor Function Classification System

**Table 5:** Intra Class Correlation Coefficient (ICC) and Standard Error of Measurement (SEM) for Inter-Rater Observer 10 MWT in Adults with Spastic CP.

	**n**	**ICC**	**95% CI of ICC**	**SEM**	**MDC**
**All participant**	30	1.15 (0.57)	0.15-2.04	1.15 (0.56)	0.15-2.08
**GMFCS** * **level I**	12	1.65 (0.34)	1.0-2.04	1.63 (0.34)	0.99-2.08
**GMFCS** * **level II**	8	1.15 (0.30)	0.58-1.45	1.13 (0.30)	0.58-1.43
**GMFCS** * **level III**	10	0.57 (0.38)	0.15-1.21	0.57 (0.37)	0.15-1.17

GMFCS*: : Gross Motor Function Classification System

## Discussion

The results show that reliability of the intra-rater (ICC= 0.98) and inter-raters (ICC = 0.99) of the 10 MWT in adults with spastic cerebral palsy is excellent. The results of this test-retest reliability study are similar to Bowden and Behrman on patients with spinal cord injury (ICC = 0.97), Collen et al. on stroke patients (0.95 <ICC <0.99) and Steffen and Seney on Parkinson’s disease (ICC = 0.97) (14,16,17). The inter-rater reliability of this study are very similar to the research of Wolf et al. on stroke patients (ICC = 0. 998) and Tyson and Connell with traumatic brain injury (ICC = 0.99) (9,25).

It seems that, the similarity of execution for all participants, same standing situation of therapists to each other’s and to the tested, simplicity and comprehensible of test and expert of therapists are reasons of high reliability. In addition, the higher ICC in the inter-raters reliability versus the intra-rater indicates individual variation of walking speed in different days. 

It can be influenced by person’s mood and motivation or environmental factors. Excellent test-retest and interraters correlation in adults with CP and among three GMFCS subgroups (ICC>0.89) indicates that this tool can be used in clinics to assess the results of interventions in adults with spastic CP, especially in mild to moderate motor impairments. The results obtained from this study are inconsistent with the reports of test-retest reliability research of Thompson et al. on children with CP (19).

The reliability of the fast 10 MWT for all participants in their reports was acceptable (ICC = 0.81). Among three subgroups of GMFCS level I, II and III the reliability was moderate to good (ICC <0.70 ). Thompson et al. interpreted that test-retest low reliability of the fast 10 MWT compared to other neurological diseases may be related to the research methodology (19). In the retest for some participants, they used another examiner. In our intra-rater research, all tests were performed by one physiotherapist. The time between our test and retest was a week, while in the study of Thompson et al. for some participants it lasted 14-days. Another difference between our and their study method was the test course. We considered two meter plus 10 meter at start and stop lines to eliminate the impact of spasticity on acceleration or deceleration. This was not the case for them. The range of participant’s age (adult versus child) is another reason of the differences in the obtained results. Despite these differences in approach, there is a similar result in terms of the reliability within the subgroup of GMFCS level III. This subgroup in our and their research had greater between-participant variability (SD<0.38). They also had higher ICC than those in levels I and II of GMFCS. It can be explained that people in GMFCS level III, due to greater influence of spasticity; joint stiffness and muscle weakness do not have ability to change the walking speed as the other subgroups of the research.

According to the calculated SEM (0.07 intra-rater and 0.02 inter-raters), probably 95%, the walking speed in the second measurement by another person or the same one twice the SEM, it will be more or less than the first walking speed measured (±0.14 in the retest and ±0.04 inter-raters). A change in subjects’ walking speed because of any therapeutic intervention greater than the MDC (calculated in this intra-rater reliability study 0.18 and between raters 0.05), most likely represents a real change that may not be attributed to measurement error. 

**In Conclusion, **the intra-rater and inter-raters reliability of the 10 MWT in adults with spastic cerebral palsy was excellent. This test is safe and cost-effective to measure walking speed in CP patients with mild to moderate motor function disorders. It is recommended to study with more samples in spastic CP, as well as in other types of CP including atetoid and ataxic.
